# Multiferroic nanocomposite fabrication via liquid phase using anodic alumina template

**DOI:** 10.1080/14686996.2018.1493888

**Published:** 2018-07-24

**Authors:** Go Kawamura, Kazuhiro Ohara, Wai Kian Tan, Taichi Goto, Yuichi Nakamura, Mitsuteru Inoue, Hiroyuki Muto, Kazuhiro Yamaguchi, Aldo R. Boccaccini, Atsunori Matsuda

**Affiliations:** a Department of Electrical and Electronic Information Engineering, Toyohashi University of Technology, Toyohashi, Japan; b Institute of Biomaterials, University of Erlangen-Nuremberg, Erlangen, Germany; c Institute of Liberal Arts and Science, Toyohashi University of Technology, Toyohashi, Aichi, Japan; d JST, PRESTO, Kawaguchi, Saitama, Japan; e Electrical and Electronic Engineering Course, National Institute of Technology, Ibaraki College, Hitachinaka, Japan

**Keywords:** Sol-gel, templating, nanotube arrays, CoFe_2_O_4_, BaTiO_3_, multiferroicity, 40 Optical, magnetic and electronic device materials, 102 Porous / Nanoporous / Nanostructured materials, 301 Chemical syntheses / processing

## Abstract

We report a novel and inexpensive fabrication process of multiferroic nanocomposite via liquid phase using an anodic alumina template. The sol-gel spin-coating technique was used to coat the template with ferrimagnetic CoFe_2_O_4_. By dissolving the template with NaOH aqueous solution, a unique nanotube array structure of CoFe_2_O_4_ was obtained. The CoFe_2_O_4_ nanotube arrays were filled with, and sandwiched in, ferroelectric BaTiO_3_ layers by a sol-gel spin-coating method to obtain the composite. Its multiferroicity was confirmed by measuring the magnetic and dielectric hysteresis loops.

## GRAPHICAL ABSTRACT

**Figure:**
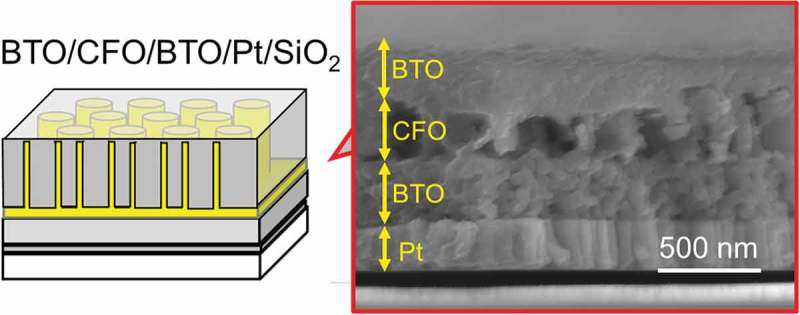

## 1. Introduction

Very few single-phase materials independently exhibit ferromagnetism and ferroelectricity; hence, multiferroic nanocomposites consisting of ferromagnetic and ferroelectric phases have been attracting much attention [1]. These nanocomposites have the potential for applications in a variety of multifunctional devices, such as data storage media, in which magnetization can be reversed by applying an electric field (or vice versa), and microwave frequency transducers where the magneto-electric coupling constant depends on the frequency of applied alternating magnetic field [2–5]. Although several attempts for fabricating bulk-type multiferroic nanocomposites, by ceramic sintering and hot molding for example, have been reported so far [6,7], much more studies on fabrication of film-type nanocomposites by sol-gel process [8,9] and pulsed laser deposition (PLD) [10–13] have been recently published.

Liu et al. [8] reported a modified sol-gel method in which a mixed precursor sol of CoFe_2_O_4_ (CFO) and Pb(Zr,Ti)O_3_ (PZT) phases was used to simplify the conventional layer-by-layer fabrication process. The prepared nanocomposite thin film contained CFO and PZT grains of 5–10 nm, which were dispersed well in the film. The ferro/ferrimagnetism and ferroelectricity of the nanocomposite film were confirmed, and the magneto-electric coupling in the film was also demonstrated in their paper. Although the reported properties are not quite impressive, the developed process is one of the simplest and most inexpensive ways to produce multiferroic nanocomposites.

In order to achieve better properties, especially strong magneto-electric coupling, Zheng et al. [10] demonstrated the advantage of a nanocomposite structure in which ferro/ferrimagnetic nanopillars are embedded in a ferroelectric matrix. They employed CFO and BaTiO_3_ (BTO) as ferrimagnet and ferroelectric, respectively, and fabricated the nanocomposite on a SrTiO_3_ (001) (STO) substrate by PLD from a single Ba-Ti-Co-Fe-oxide target via self-assembly. The nanocomposite showed a strong magneto-electric coupling through magnetostrictive and piezoelectric properties of the two phases. This type of nanostructure containing nanopillars of ferro/ferrimagnet has then been further investigated by other researchers. Schmitz-Antoniak et al. [12] have analyzed the strain-mediated coupling between the two subsystems, CFO and BTO. They demonstrated that an in-plane magnetic field broke the tetragonal symmetry of the (1,3)-type CFO/BTO structures. This phenomenon was discussed in terms of off-diagonal magnetostrictive-piezoelectric coupling, which creates staggered in-plane components of the electric polarization. Ross et al. [13,14] developed a process for combinatorial PLD using a self-made ultrapure target for each component. They prepared a variety of samples with different periodicities and compositions. For instance, Co*_x_*Ni_1-*x*_Fe_2_O_4_/BiFeO_3_ (BFO) nanocomposites (0 ≤ *x* ≤ 1) were fabricated on Nb-doped STO substrates, and the magnetostatic interactions among Co*_x_*Ni_1-*x*_Fe_2_O_4_ nanopillars were controlled well by altering *x*.

As demonstrated in the previously mentioned references, the nanocomposite structure, in which ferro/ferrimagnetic nanopillars are embedded in a ferroelectric matrix, has superior multiferroic properties, especially the magneto-electric coupling. However, only gas-phase syntheses have been employed to fabricate such structures. In this work, we report a liquid-phase fabrication approach for nanocomposites with ferromagnetic nanotube arrays embedded in a ferroelectric matrix. This structure resembles the nanopillar arrays arrangement of a ferromagnet in a ferroelectric matrix, and thus a strong magneto-electric coupling is expected. We spin-coated an anodic alumina oxide template (AAO) with CFO solution to fabricate CFO nanotube arrays, which were then embedded in a BTO matrix. Ferromagnetism and ferroelectricity of the nanocomposite were individually confirmed, indicating that the nanocomposite possesses multiferroicity.

## 2. Experimental details

Commercial AAO (ultrathin type; horizontal size: 15 × 15 mm, thickness: 270–320 nm, pore size: 500 nm, pore-pore distance: 450 nm; TopMembranes Technology, China) was purchased and used for the following experiments as a template. As-received AAO on a poly(methyl methacrylate) (PMMA) substrate was first transferred to a SiO_2_ substrate by dissolving PMMA with acetone for the following heat-treatment process.

In preparation of 0.2 M CFO precursor solution, 4.85 g of Fe(NO_3_)_3_ · 9H_2_O (99.0%; Wako, Japan) was dissolved in 21 mL of C_3_H_8_O_2_ (2-methoxyethanol) (99.0%; Wako, Japan). After complete dissolution, 1.49 g of Co(CH_3_COO)_2_ · 4H_2_O (99.0%; Wako, Japan) was added to the solution and stirred until it was completely dissolved. Finally, 9 mL of CH_3_COOH (acetic acid) (99.9%; Wako, Japan) was added to adjust the concentration of CFO. The nanotube arrays of CFO on a SiO_2_ substrate were prepared by means of spin-coating of AAO with the CFO precursor solution. The CFO precursor solution was dropped manually onto an AAO/SiO_2_ substrate; then, it was spun at 5000 rpm for 30 s. During spinning, 2-methoxyethanol was sprayed onto the substrate to remove excess amount of CFO solution from the top surface of the AAO template. A detailed description of this spraying process is reported elsewhere by our group [15]. After drying it on a hot plate set at 150 °C for 10 min, the substrate was pre-annealed at 400 °C for 10 min. This CFO coating process was repeated three times to increase the amount of CFO in the tubular pores of AAO template. The CFO/AAO on a SiO_2_ substrate was annealed at 800 °C for 1 h; then, the AAO template was completely dissolved with 0.03 wt% NaOH solution.

As for preparing 0.5 M BTO precursor solution, 6.38 g of Ba(CH_3_COO)_2_ (99.0%; Wako, Japan) and 15 mL of acetic acid were mixed and stirred at 60 °C until they were completely mixed. Separately, 7.44 mL of Ti(OC_3_H_7_)_4_ (95.0%; Wako, Japan) was mixed with 7.44 mL of C_5_H_8_O_2_ (acetylacetone) (99.0%; Wako, Japan) and stirred at room temperature. Then, the solutions were mixed and added to 25.12 mL of 2-methoxyethanol, followed by mixing with 2 mL of H_2_O to adjust the concentration.

The multiferroic nanocomposite film, BTO/CFO (nanotube)/BTO, was prepared on a Pt coated SiO_2_ substrate, where Pt was first coated on a SiO_2_ substrate by ion-beam sputtering for the following dielectric property measurement. The substrate was spin-coated with 0.5 M BTO precursor solution at 3000 rpm, followed by drying at 100 °C for 10 min and pre-annealing at 150 °C for 30 min. This process was repeated twice to obtain a thick BTO layer. An AAO template was transferred onto the BTO flat layer for the next step. The CFO precursor solution was used for spin-coating of the AAO-placed substrate at 5000 rpm for 30 s. During spinning, 2-methoxyethanol was sprayed on the substrate to remove excess CFO from the top surface of the AAO template. Drying and pre-annealing were carried out at 150 °C for 10 min and 400 °C for 10 min, respectively. This CFO coating process was repeated three times; then, the sample was annealed at 800 °C for 1 h. The AAO template was dissolved by immersing the sample into 0.03 wt% NaOH solution for 24 h. The sample was spin-coated again with 0.2 M BTO precursor solution at 5000 rpm, followed by drying at 100 °C for 10 min and pre-annealing at 150 °C for 30 min. This BTO coating process was repeated twice. Finally, the sample was annealed at 800 °C for 1 h. As for the dielectric property measurement, a Ag paste was applied on a ~1 mm^2^ circled area to act as the upper electrode.

The samples were observed with a scanning electron microscope (SEM, Hitachi S-4800, Tokyo, Japan) and a transmission electron microscope (TEM, JEOL JEM-2100F, Tokyo, Japan). Elemental distributions were analyzed by means of scanning transmission electron microscopy combined with energy dispersive X-ray spectroscopy (STEM-EDX, JEOL-2100F, and JEOL 2300T, Tokyo, Japan). An X-ray diffractometer (XRD, Rigaku Ultima IV, Tokyo, Japan) was used to analyze the crystal structure of the samples. The magnetic hysteresis loops of the samples were recorded with a vibrating sample magnetometer (VSM, Tamakawa, TM-VSM261483-HGC-SG, Miyagi, Japan). The dielectric constants as a function of frequency were measured using a potentiostat (Solartron Analytical (AMETEK), 1287A, Berwyn, PA, USA). The dielectric hysteresis loops of the samples were recorded with a dielectric property evaluation system (Toyo Corporation, 6252Rev.C, Tokyo, Japan).

## 3. Results and discussion

### 3.1 CFO nanotube arrays

**Figure 1.**
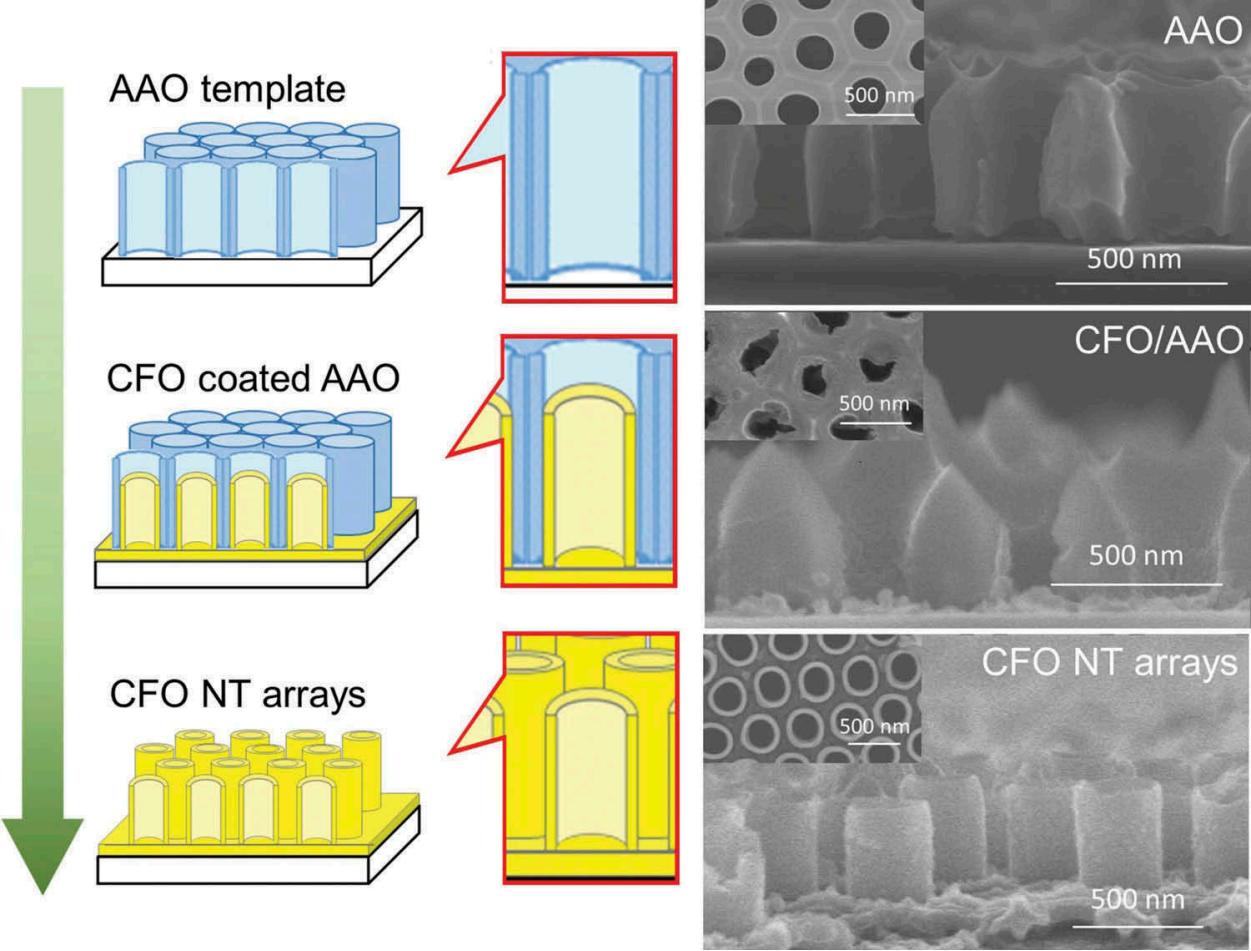
Schematic of the fabrication process for CFO nanotube arrays on a SiO_2_ substrate. The corresponding SEM images are shown to the right. From top to bottom: AAO template on a SiO_2_ substrate; CFO coated AAO on SiO_2_; CFO nanotube arrays on SiO_2._

**Figure 2.**
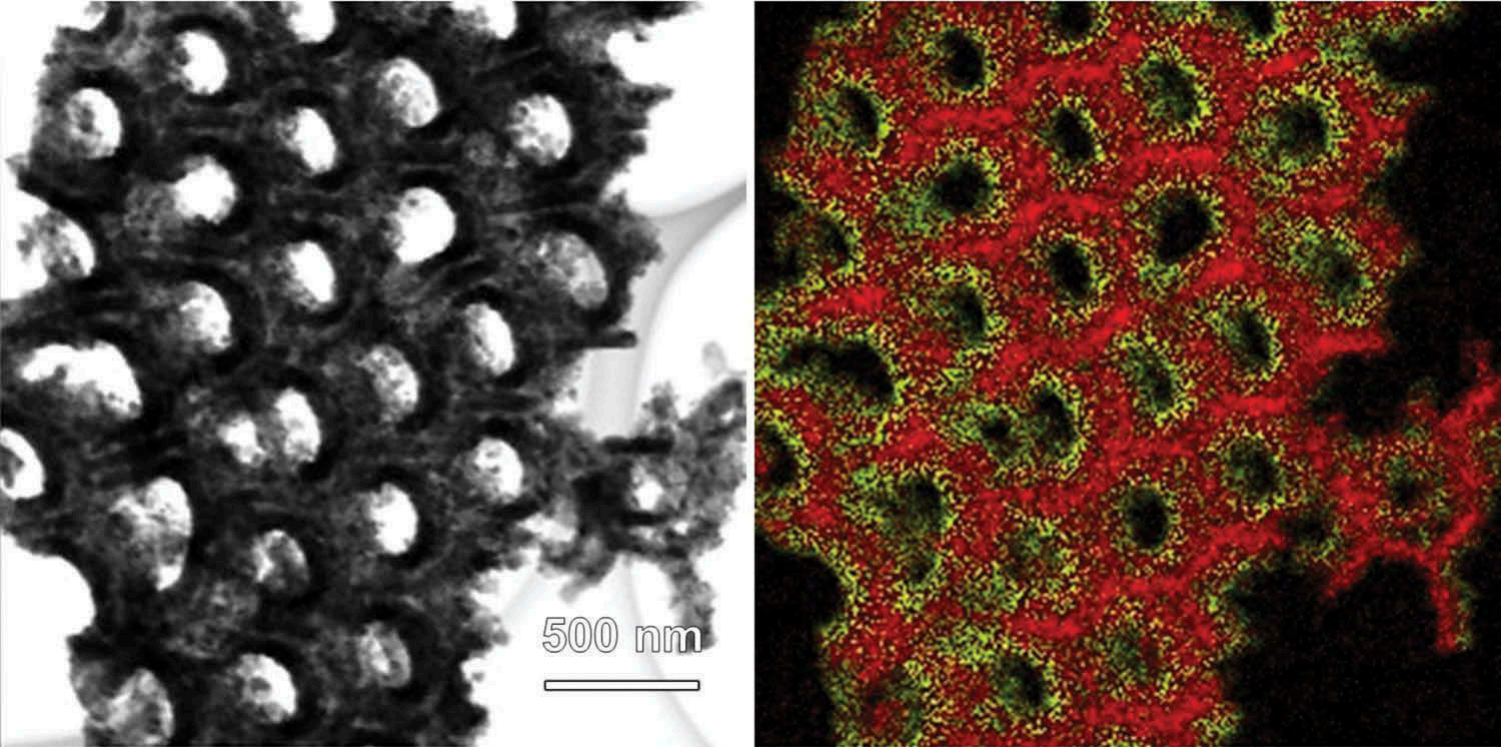
STEM (left) and the corresponding EDX images (right) of CFO coated AAO. In the EDX image, red and yellow colors represent Al and Co elements, respectively.

The CFO nanotube array fabrication process in this work is summarized in Figure 1. First, CFO was deposited on the walls of the AAO template by spin-coating of AAO/SiO_2_ with the CFO precursor. The CFO was observed both on the SiO_2_ surface and AAO walls, because of the tiny gaps between the AAO template and the SiO_2_ substrate. Because 2-methoxyethanol was sprayed on AAO during spin-coating process, there was no CFO on the top surface of AAO. In contrast, a CFO compact layer was formed on the top surface of AAO when the process of 2-methoxyethanol spray was skipped (the image is not shown). The structure of the CFO coated AAO was further studied by STEM-EDX (Figure 2) in samples where the CFO-coated AAO was scratched off the SiO_2_ substrate for the observation. It was confirmed that the inner walls of AAO were homogeneously coated with CFO without closing the tubular pores of AAO. Finally, CFO nanotube arrays on a SiO_2_ substrate were obtained by dissolving the AAO template with NaOH. For the complete removal of the AAO template without collapsing the nanotube array structure of CFO, immersion of the sample into 0.03 wt% NaOH for 24 h was found to be the best condition.

**Figure 3.**
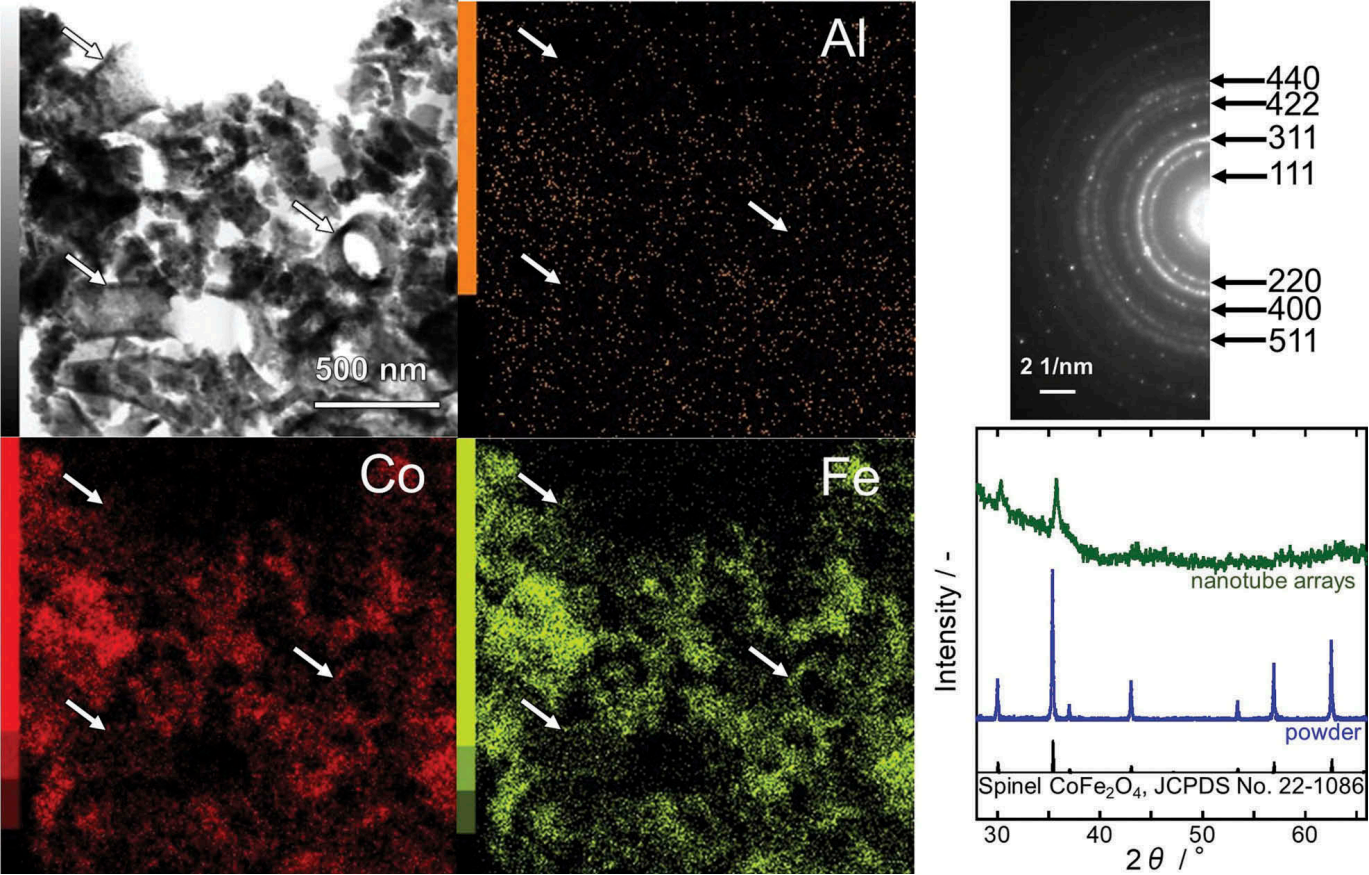
STEM (upper left), the corresponding EDX images (center and lower left) and SAED pattern (upper right) of CFO nanotubes. The XRD patterns of the CFO nanotube arrays on a SiO_2_ substrate and of CFO powder are shown at the lower right.

The CFO nanotube arrays obtained via the process described in Figure 1 were scratched off from the SiO_2_ substrate and observed with STEM-EDX (Figure 3, left). Although the majority of CFO nanotubes were collapsed by the process of scratch-off because of their mechanical weakness, some of them survived and could be observed in the STEM-EDX images (indicated by white arrows). The EDX analyses confirmed that the sample was composed of only three elements, Co, Fe, and O, and thus, the complete removal of Al by the NaOH treatment was proven. The selected-area electron diffraction (SAED) pattern (Figure 3, upper right) indicated that the sample consisted of a single phase of spinel CFO. In order to further verify the crystal structure of the sample, an XRD pattern was recorded (Figure 3, lower right). However, the signal-to-noise ratio of the pattern was too low because of the low amount of CFO on the SiO_2_ substrate. Therefore, the CFO precursor solution was poured into a petri dish and annealed at 800 °C for 1 h after drying in order to obtain a CFO powder sample. The XRD pattern of the powder sample perfectly matched the reference data (JCPDS No. 22–1086). No secondary phase was detected, indicating that the nanotube sample was also entirely composed of spinel CFO.

### 3.2 Multiferroic nanocomposite

**Figure 4.**
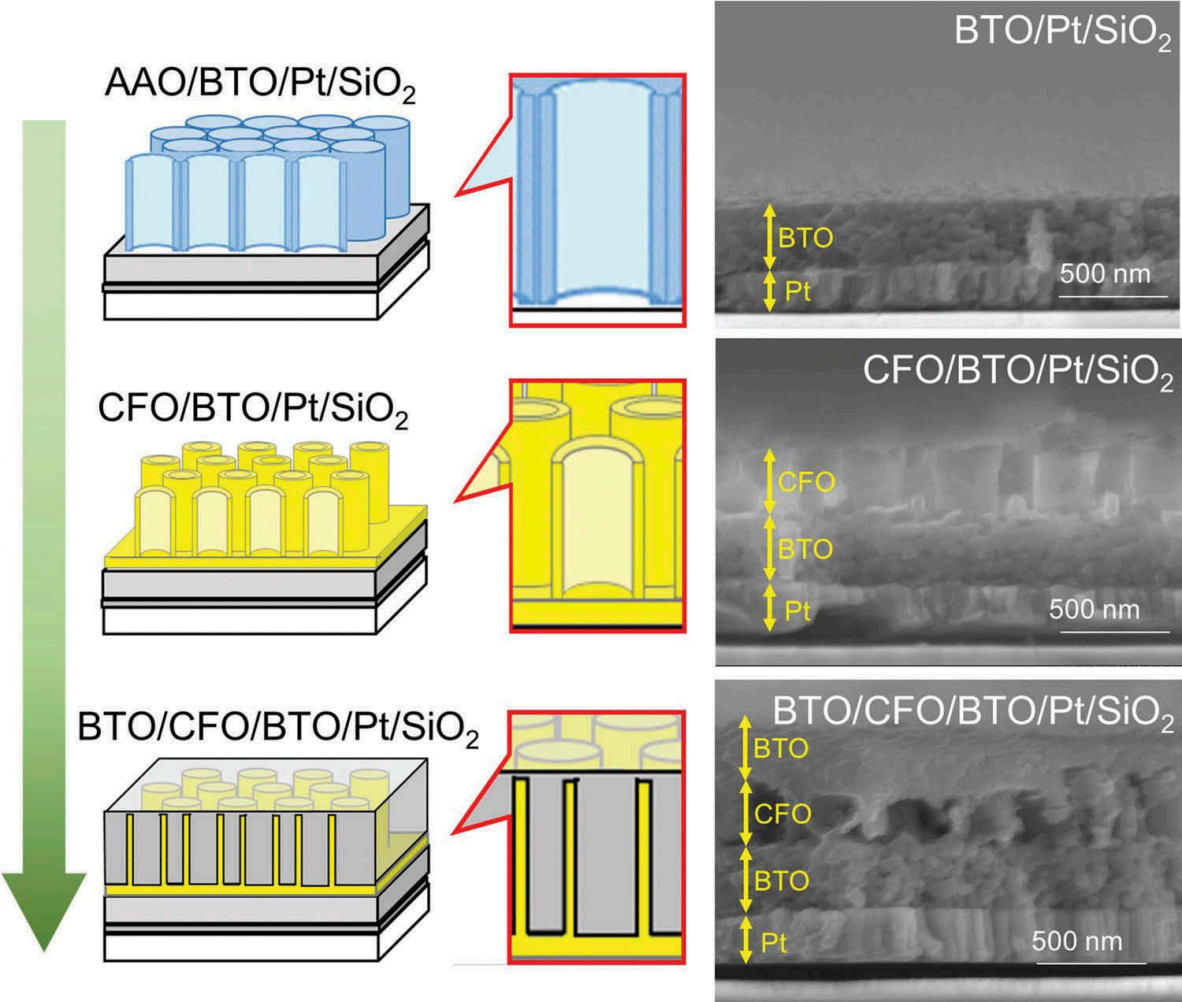
Schematic of the fabrication process for multiferroic nanocomposite, BTO/CFO/BTO, on a Pt-coated SiO_2_ substrate. The corresponding SEM images are shown to the right. From top to bottom: (AAO-placed) BTO compact layer on a Pt-coated SiO_2_ substrate; CFO nanotube arrays on BTO/Pt/SiO_2_; BTO coated CFO nanotube arrays on BTO/Pt/SiO_2._

A multiferroic nanocomposite was then fabricated by modifying the process for CFO nanotube arrays. The overall fabrication process is summarized in Figure 4. The thickness of CFO nanotube array layer was fixed to be around 250 nm in this work due to the fixed dimension of the AAO template. Therefore, a BTO compact layer must have been fabricated for the dielectric property measurement; otherwise, high electric fields could not be applied to the sample. For this reason, a BTO compact layer was first coated on a Pt electrode-sputtered SiO_2_ substrate. Then, CFO nanotube arrays were fabricated on the BTO layer using the same process shown in Figure 1. Finally, the CFO nanotube arrays were spin-coated with BTO precursor solution without cleaning the BTO layer formed on the top of CFO nanotube arrays; hence, a compact BTO layer was also formed on the top of the CFO nanotube layer as well as on the bottom of the CFO layer. As can be seen in the SEM image of BTO/CFO/BTO/Pt/SiO_2_ (Figure 4, lower right), the CFO nanotube array layer was not fully filled with BTO, meaning that the nanocomposite film had porous structure, which would strongly affect the dielectric and multiferroic properties of the film.

**Figure 5.**
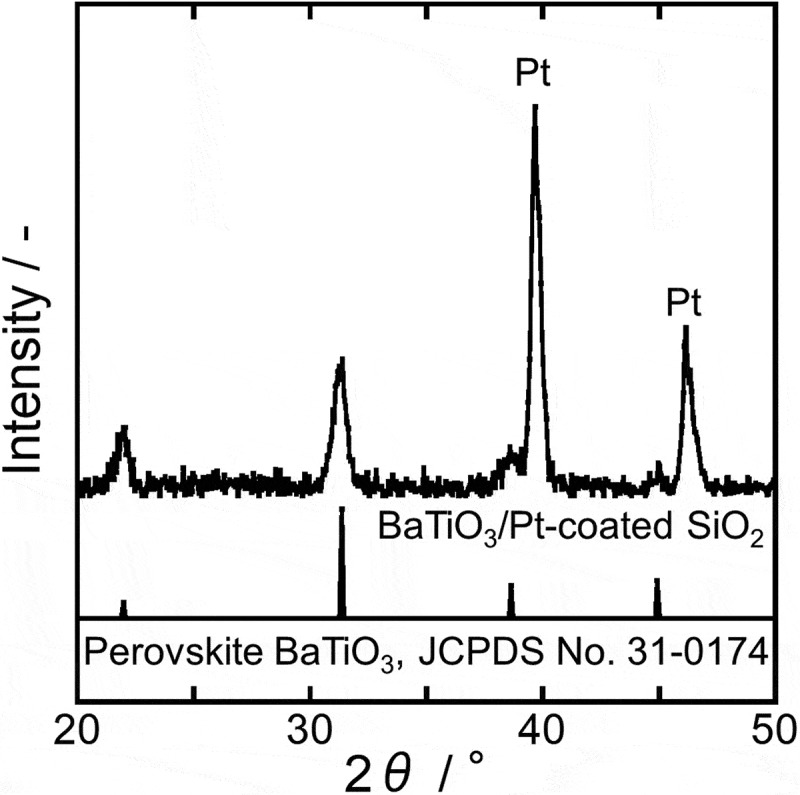
XRD pattern of the BTO compact layer on a Pt-coated SiO_2_ substrate.

The XRD pattern of the prepared BTO layer is shown in Figure 5. Because the CFO amount in the BTO-CFO nanocomposite was too small to be detected by XRD, only the BTO peaks are visible. Crystallization of single-phase perovskite BTO was confirmed by comparing this XRD pattern to the reference data (JCPDS No. 31–0174). Because the formation of spinel CFO in the CFO layer was confirmed by the XRD pattern of CFO NT arrays in Figure 2, the obtained nanocomposite should consist of ferrimagnetic spinel CFO nanotube arrays embedded in a ferroelectric perovskite BTO matrix, suggesting that the nanocomposite should exhibit multiferroicity.

**Figure 6.**
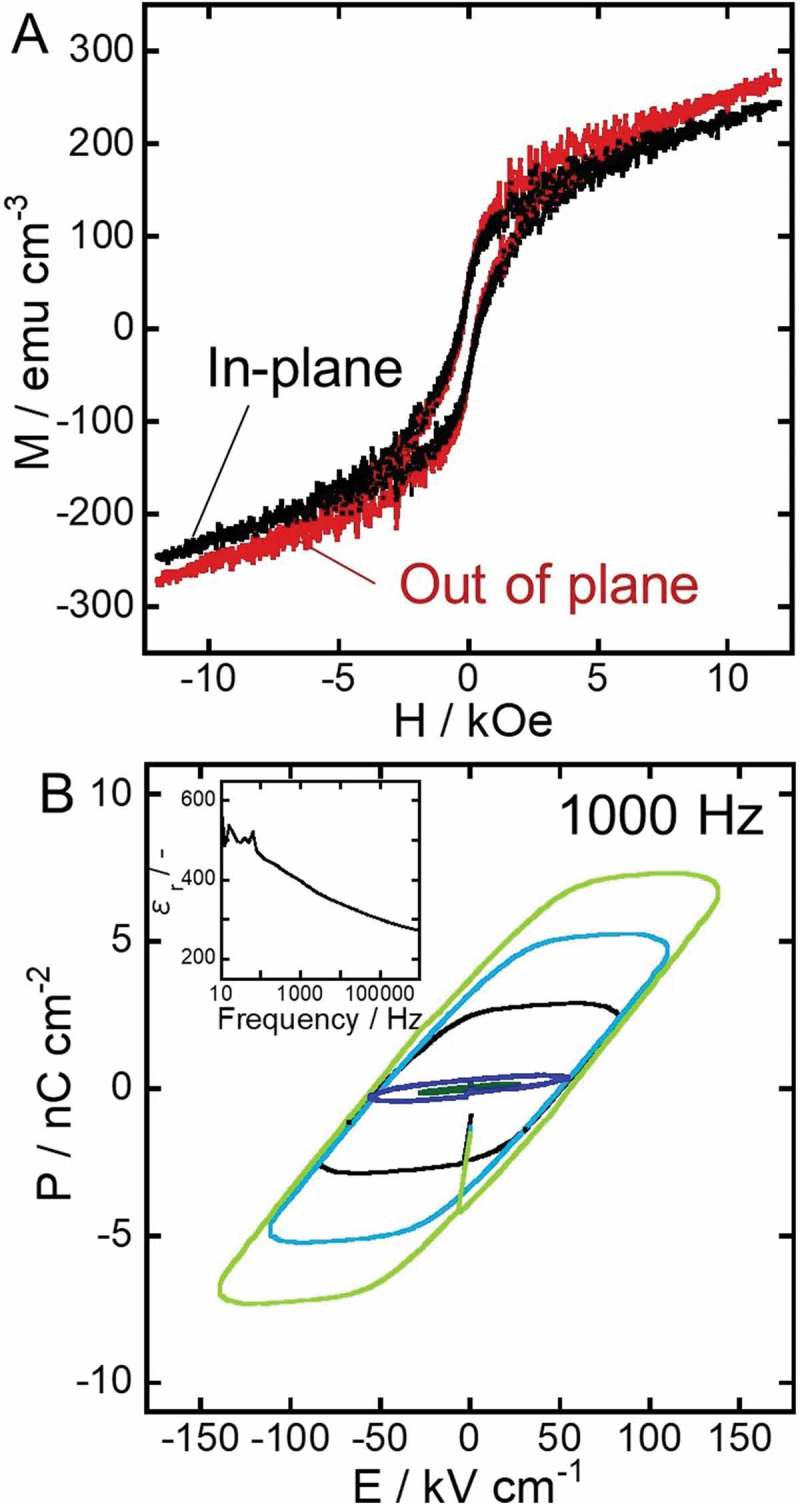
Magnetic (A) and dielectric (B) hysteresis loops of the multiferroic nanocomposite. The inset of the panel B shows the relative permittivity versus frequency.

To verify the multiferroic property of the new nanocomposites, the magnetic and dielectric hysteresis loops were recorded and are shown in Figure 6. The saturation magnetization and coercivity evaluated from the magnetic hysteresis loops in Figure 6(a) were about 150 emu cm^−3^ (normalized to the volume fraction of CoFe_2_O_4_, ~ 20%) and 350 Oe, respectively, regardless of the orientation of the applied magnetic field. In comparison with similar reports [10,16,17], the out of plane coercivity of our sample is relatively small. This behavior can be explained by the following reasons:

1. the anisotropy is intrinsically small because the CFO nanotubes are short (length: ~250 nm, diameter: ~500 nm)
2. diffusion or rearrangement of atoms would occur at the interface of CFO and BTO, resulting in the deterioration of the magnetic properties of CFO
3. the vicinity of superparamagnetic limit should also be considered in our case because the walls of CFO nanotubes are very thin. As known, superparamagnetism appears because magnetization direction of each crystallite smaller than 8 nm can be randomized by thermal excitations at room temperature, resulting in no coercivity [18]

Therefore, the out of plane, coercivity could be enhanced by growing longer CFO nanotubes and/or increasing the thickness of the nanotubes’ wall. Nevertheless, magnetic hysteresis was observed in both in-plane and out-of-plane measurements, proving that our CFO-BTO nanocomposite exhibited ferrimagnetism. The coexistence of ferrimagnetism and nano-periodic structure in the nanocomposite would lead to magnetostatic interactions among CFO nanotubes [13,14].

The relative permittivity of the sample was altered from 500 to 300 when the frequency was changed from 10 to 10^6^ Hz, as shown in the inset of Figure 6(b). Thus, in this work, a frequency of 1000 Hz was employed for recording dielectric hysteresis loops because stable polarization was expected during the measurement. The hysteresis loops in Figure 6(b) show the linear relationship between the applied voltage (30–150 kV cm^−1^) and polarization, and the remanent polarization of ~3.8 nC cm^−2^ at ~150 kV cm^−1^. This value of remanent polarization was quite small compared to previously reported works [19–22]. The major reason of the small value obtained in this work is likely the unexpected porous structure of the nanocomposite. More specifically, thin strips of BTO coating formed in the nanocomposite due to incomplete BTO filling of the CFO nanotubes could lead to superparaelectric limit, resulting in the weak polarization of the sample. Similar to superparamagnetism, superparaelectricity can be induced in ferroelectric nanoparticles and thin films, for which the energy to coherently flip the polarization vector becomes comparable to the thermal excitation energies [23]. In order to increase the remanent polarization, complete filling of CFO with BTO will be crucial, which also enables a poling process at huge poling voltage. As for achieving the absolute filling of nanotubes, supercritical fluid impregnation [24], vacuum impregnation [25,26], electrophoretic deposition of BTO nanoparticles [27], and the combination of them will work; thus, they are currently under investigation in our research group. On the other hand, BTO nanoparticle assembly has shown enhanced dielectric properties compared to bulk BTO [28]. Moreover, a possibility of drastic modulation of Curie temperature and piezoelectricity with controlling the pore structure of BTO has recently been reported [29]; thus, the property improvement of our nanocomposite can be well expected. Although the remanent polarization was still very small, ferroelectricity of the nanocomposite was confirmed from the clear dielectric hysteresis loops in Figure 6(b). Therefore, it was proven that the fabrication process introduced in this work was useful to produce a multiferroic nanocomposite with novel nanostructure.

Although ferrimagnetism and ferroelectricity (multiferroicity) were separately demonstrated in our BTO/CFO nanocomposite, a clear magneto-electric coupling was not observed when the dielectric properties were measured with and without an external magnetic field at 100–3000 Oe (data not shown). This is presumably because the nanocomposite contains undesired variant pores, which work as hindrance to the strain-mediated magneto-electric coupling. Therefore, complete filling of the pores by the previously mentioned methods or controlling the porous structure formation before the appropriate poling and magnetization treatments will result in an effective magneto-electric coupling.

## 4. Conclusions

This work demonstrated that the sol-gel spin-coating method, by using an AAO template, was a low-cost and useful approach to the fabrication of multiferroic nanocomposites consisting of ferrimagnetic CFO nanotube arrays embedded in a ferroelectric BTO matrix. 2-methoxyethanol spray cleaning during spin-coating of AAO with the CFO precursor solution removed the CFO layer from the top surface of AAO, resulting in the formation of CFO nanotube arrays on a substrate. The AAO template had to be dissolved very slowly, within 24 h, in order to maintain the nanotube array structure of CFO. The single-phase crystalline structures of spinel CFO and perovskite BTO were confirmed in CFO and BTO layers, respectively. Although the measured out of plane coercivity and remanent polarization were relatively small, the multiferroicity of the sample was confirmed from the recorded magnetic and dielectric hysteresis loops. This work will hopefully motivate further studies on nanocomposite fabrication using low-cost liquid-phase processes rather than expensive gas-phase technologies.
